# Additive Manufacturing for Localized Medical Parts Production: A Case Study

**DOI:** 10.1109/ACCESS.2021.3056058

**Published:** 2021-02-01

**Authors:** Victor Verboeket, Siavash H. Khajavi, Harold Krikke, Mika Salmi, Jan Holmström

**Affiliations:** 1 Brightlands Institute for Supply Chain InnovationZuyd University of Applied Sciences–Maastricht 6200 Maastricht The Netherlands; 2 Department of Industrial Engineering and ManagementAalto University 174277 02150 Espoo Finland; 3 Faculty of Management SciencesOpen University of the Netherlands 6401 Heerlen The Netherlands; 4 Department of Mechanical EngineeringSchool of EngineeringAalto University 174277 00076 Espoo Finland

**Keywords:** Additive manufacturing, medical parts, localized manufacturing, cost modeling

## Abstract

Centralized supply chains (SCs) are prone to disruption, which makes them a risky choice for medical equipment production. Additive manufacturing (AM) allows for production localization and improvements in SC resilience. However, the comparative competitiveness of a localized SC from the time and cost perspective is still unclear. In this study, we investigate the competitiveness of localized medical part AM SCs against centralized ones by analyzing the responsiveness and cost of each SC. We utilize a real-world case study in which an AM service provider supplies medical parts to university medical centers in the Netherlands to construct six scenarios. We also develop a thorough empirical cost formulation for both central and local AM of patient-specific medical parts. The results of scenario analysis show that when utilizing the currently available AM technology, localized SC configurations significantly reduce the delivery time from about 54 to 27h, but at a 4.3-fold higher cost. Hence, we illustrate that the cost difference between the localized and centralized scenarios can be reduced when state-of-the-art AM machines are utilized, demand volumes increase, and the distances between the SC network nodes expand. Moreover, our scenario analysis confirms that the cost of the measures taken to prevent dust dispersion associated with powder-bed fusion AM has a major impact on the total cost of localized AM SCs for medical parts. The results of this study contribute to the understanding of the relevant factors in deciding whether central or localized SC configurations can be used in the AM production of medical parts. Furthermore, this study provides managerial insights for decision-makers at governments and hospitals as well as AM service providers and AM equipment manufacturers.

## I. Introduction

Centralized supply chains (SCs) are among the main sources of efficiency and cost-effectiveness in today’s global economy. However, events such as pandemics and natural disasters have illustrated the weaknesses of centralized SC designs [1]. In 2011, during Thailand’s floods and Japan’s earthquake and tsunami, major disruptions occurred in the SC of items such as computer hard disk drives and automotive parts, respectively. A similar but larger impact was registered during the early stages of the COVID-19 pandemic in 2020, which impacted the availability of medical parts, including products that are critical in the fight against the pandemic, such as test swabs and personal protective equipment [2]. In such situations, flexibility in the manufacturing and SCs proves to be very valuable [3]. In some cases, SC flexibility is developed using local digital manufacturing solutions [4]. In this study, we explore the justification and feasibility of such localization decision for additive manufacturing (AM).

AM is a direct digital manufacturing technology in which material is added layer by layer, in contrast to traditional (subtractive) manufacturing, in which material is gradually removed from an initial block to achieve the intended shape [5]. In general, AM allows for the production of complex geometries and mass customization without incurring additional costs. In [6], the authors describe how AM can be disruptive in one context while causing only minor changes in another, whereas in [7], [8], AM is viewed as useful for particular applications. In [9], several examples of potentially disruptive effects on SCs are provided, for instance, when manufacturing in low-wage countries moves from factories to households. Generally, AM is suitable for *in situ* (local) manufacturing since digital files easily bypass physical boundaries [10], [66]. During the early phase of the COVID-19 pandemic, AM was used locally to alleviate the scarcity of nasal test swabs, confirming the risks related to centralized manufacturing SCs for basic medical parts. The conventional manufacturing of nasal test swabs was centralized in Italy, thus causing severe difficulties to ramp up testing in different countries worldwide and, hence, worsening the situation with regard to the pandemic. According to [11], localized AM supported the alleviation of the shortage of swabs for testing, illustrating the use of AM for SC resilience improvement.

AM is used in many medical applications, such as preoperative models, implants, tools, and instruments [12]. Some of these applications are still in the research phase, but others are already commercially available. In line with [13], in which it was mentioned that the use of direct digital prototyping may accelerate and simplify in-house design, several university medical centers (UMCs) in the Netherlands have sought to improve the treatment quality. Increasing the speed of feedback, as a result of switching from centralized to localized AM, of patient-specific medical care products helps improve the “experiential learning” [14]. In this study, we investigate the situation in different UMCs throughout the Netherlands to understand the impact of localized AM SCs on the responsiveness of medical parts and economics of such implementation. The findings provide relevant managerial implications in guiding the preparation efforts before and during a pandemic.

The remainder of this article is organized as follows. In Section II, we review the current literature. In Section III, we describe the research methodology, followed by the results in Section IV, a discussion in Section V, and the conclusions in Section VI.

## II. Literature Review

This section introduces the relevant SC concepts and examines those related to AM. It also explains the effects of AM on SC design and defines the gap in the literature.

### A. Supply Chain Management

Supply chain management (SCM) is a management field that has originated in logistics [15]. While logistics mainly focuses on the flow of materials, SCM takes a more holistic approach and includes the flow of information and money. With the emergence of the supply chain operations reference (SCOR) model [16], SCM definitions have been standardized, thus simplifying the benchmarking of processes and performances. In general, the performance of an SC is measured from its outcomes. In this study, we focus on the traditional SC outcomes of *cost* and *responsiveness* according to the SCOR definitions. Costs are defined as the total SC management costs (i.e., the sum of the costs associated with planning, sourcing, making, delivering, and returning orders). Responsiveness is defined as the order fulfillment cycle time, the time from order receipt to order acceptance [16].

Having a well-designed SC is strategically vital for a firm’s competitiveness [9]. Each SC design comprises choices related to manufacturing, inventory, assortment, personnel, information and communications technology, and transportation modalities [17]. In this study, we explore one of the most important decisions in SC design: when to switch from centralized to localized SC configurations.

SC designs are heavily influenced by technological and societal changes. According to the World Economic Forum, society is on the verge of the fourth industrial revolution [18], whereby increasingly fast computing, for instance, mobile supercomputers and artificial intelligence (AI), is revolutionizing civilizations. Computer technology is increasingly penetrating and digitizing SCs. In [19], a digital SC was defined as “a smart and value-driven process that generates business value with analytical processes and technology.” In addition to AM, digital SCs can include any other digital technology, for instance, AI, blockchain technology, Internet of Things, automated guided vehicles, or combinations of technologies, for instance, in the context of a “digital twin” [20].

### B. Additive Manufacturing and Supply Chain Impact

AM is a collection of techniques that can, depending on the material and specific technology chosen, be categorized in different ways [5]. In this study, we focus on selective laser sintering (SLS), which is a powder-bed fusion (PBF) AM technique. AM is also known as rapid prototyping (RP), rapid tooling (RT), rapid manufacturing (RM), and three-dimensional (3D) printing. After the invention of AM, prototyping (RP) applications were the first to appear in the 1980s. As more materials became printable, tooling (RT) applications followed in the late 1980s. In [21], RP and RT are considered as fully mature applications, which is in contrast to the RM of final products. Nevertheless, various use cases for the AM of final parts already exist, for instance, for industrial spare parts [22], [23], for patient-specific medical applications [24], [25], for consumer products [26], [27], and for art, hobbies, jewelry, and fashion [28].

AM has several benefits over subtractive manufacturing, although there are also potential downsides to this process. Such benefits include the potential integration of functions into the design (e.g., cooling channels), or part consolidation while benefiting from a less restrictive design for manufacturability requirements. The benefits of AM also include the absence of tools in the production process, which enables the production of very small batches of parts. Moreover, because of its layer-wise process, AM can significantly reduce raw material waste, particularly in metal PBF AM, compared to conventional manufacturing methods, such as computer numerical control machining. The downsides of AM include the fact that the production speed may be low, the machines and materials may be expensive, the automation levels during the pre- and postprocessing of 3D-printed parts may be low, and the surface finish of the parts may be poor. Moreover, no standards have yet been fully developed for AM, which limits its utilization in different industries, such as nuclear powerplants [29]–[32].

It should be noted that the ability of AM to produce any desired shape makes it suitable for personalized medical products [33], [34]. For example, it has been shown that the availability of a digital file can improve the replicability of personalized hearing aids [35], preoperative models can be used as a communication tool to improve trust and consent [25], [36], implants can speed up surgeries [37], and in-operating-room on-demand AM of coronary stents can significantly reduce the SC lead time [38].

It should also be noted that the unique characteristics of AM change the requirements for SCs while potentially improving the SC performance [39]. Compared to traditional (subtractive) manufacturing, AM may make SCs shorter and simpler, thus reducing the number of SC steps [40], [41], [42], and digital file distribution may largely replace the distribution of goods [10], [43], [7]. Moreover, the traditional design-build-deliver arrangement may shift [44].

In general, SC configuration decisions consist of the positioning of AM in the SC [45]. Centralized configurations do exist [46], [47], [48], as well as localized AM configurations, which are also called “distributed,” “*in situ*,” or “decentralized” configurations [13], [26]. Centralized design combined with localized manufacturing has also been mentioned in [35], in contrast to [49], in which localized scanning was combined with centralized manufacturing. In [46], centralized coordination was combined with localized manufacturing, whereas in [50], centralized control with different levels of localized automation was described. However, only one of these studies contributes to the SC design analysis of medical parts [24].

In [24], [51], the authors studied the implementation of AM in localized SC configurations. Localized AM may improve responsiveness (speed), yielding, for example, a shorter repair time, shorter time to market, and faster product availability [43], [51], [52]. For the SCs of medical parts, a significant reduction of the SC lead time is envisioned [35], [38]. Localized AM may reduce inventory (finished goods) and transportation costs [49], [53]. However, compared to the centralized solutions offered by AM service providers, localized locations have no economies of scope in terms of equipment utilization, raw material purchasing, labor, and knowledge [7], [23].

### C. Gap in the Literature

Recently, there has been an increase in the academic literature on the effects of AM on SC designs [7], [42], [51], [54], [55]. For example, the study in [45] describes the centralized versus localized positioning of AM in SCs and the related cost and responsiveness tradeoffs as a key research topic in SC design. In [51], [54], the authors investigated central versus localized configuration decisions regarding spare parts’ SCs. In [24], the authors investigated the economic feasibility of switching from traditional manufacturing to *in situ* AM of biomedical implants.

Among research articles on the use of AM in medical applications, only a few have utilized real-world case study material (see, e.g., [24], [35]). However, these articles are generally exploratory in nature or they compare AM with traditional manufacturing and do not compare SC configuration decisions within an already existing AM SC design. The aim of this study is to contribute to the filling the gap of knowledge regarding central versus localized medical SCs by studying a real-world SC in which an AM service provider supplies patient-specific medical care products to UMCs. Our contribution is, hence, to provide insights into the key decision-making factors related to switching from a centralized to a localized SC design configuration, considering the tradeoffs in SC responsiveness and cost performance. This research will help inform decision-making and provide opportunities for interventions at medical centers, AM service providers, and AM equipment manufacturers. The following are the research questions (RQs) that we aim to answer in this study:

RQ1: What is the comparative competitiveness of centralized versus localized AM SC configurations for patient-specific medical parts from the viewpoints of responsiveness and cost?
RQ2: How does the comparative competitiveness of centralized versus localized AM SC configurations change when technology improves, demand volume changes, and/or service distance increases?

## III. Methodology

**FIGURE 1.**
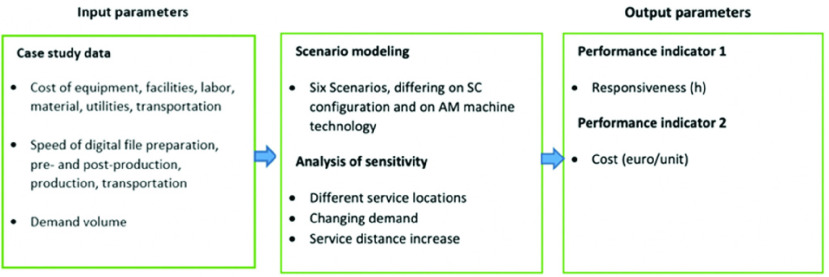
Overall methodological design.

In this study, we use data acquired from a real-world case study (explained in Section III.A) and scenario analysis (explained in Section III.B) for evaluating the impact of current and future AM machines on the SC responsiveness and cost of centralized and localized patient-specific medical parts’ SCs. Our methodological design is summarized in Fig. 1.

### A. Case Study

In [56], case study research is defined as “an empirical inquiry that investigates a contemporary phenomenon (the case) in depth and within its real-world context.” In this study, we followed the guidelines of [56] for data collection to ensure research rigor. We selected AM cases of patient-specific medical parts to contribute to the body of knowledge regarding the SC design for medical applications. According to [56], cases should be analytically generalizable, and this case represents a wide class of AM applications in a medical context.

**FIGURE 2.**
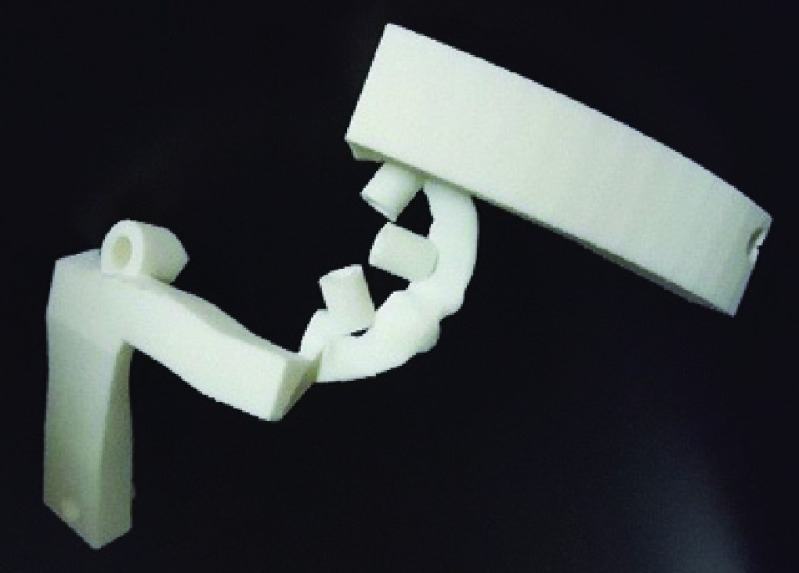
Example of a surgical guide.

In this study, we investigate the SCs of patient-specific surgical guides, which are used with cutting or drilling tools and allow for preoperative planning (Fig. 2). These personalized guides enable surgeons to plan the exact positioning for cutting and drilling the patient’s bones prior to the actual surgery. In general, being able to personalize these guides means that an improvement can be achieved in terms of surgery quality and first-time fit. Besides improving the patient’s wellbeing, this may save up to 1 hour of operating room (OR) time. In general, AM is very suitable for manufacturing such complex shapes and has been used for this purpose for several years now. A set used during surgery can consist of multiple pieces. Currently, the SC is comprised of eight UMCs located in the Netherlands, where these surgeries are performed. These UMCs are supplied by one (central, outsourced) AM service provider, also located in the Netherlands. This AM service provider uses EOS P100 AM machines, which employ the polyamide SLS PBF process [57]. Being the legal manufacturer, the UMC innovation lab uses computed tomography scan Digital Imaging and Communications in Medicine data to design surgical guides and then forwards the files for manufacturing to the AM service provider. After the manufacturing process, the lab receives the manufactured part, checks its quality, sterilizes it, and hands it over to the physician for surgery.

Our case study research started with the selection of input parameters to calculate the cost and responsiveness. Data were collected from one of the UMCs interested in localizing the AM of these surgical guides. To achieve such a localized SC configuration, each UMC requires on-site AM production.

The data collection process followed the approach outlined in [56], using logical models and time study maps for analyzing case study evidence. To make sure data were collected on all the relevant processes, an interview question protocol was developed [56]. For each SC step, data on the volume, time, and cost were gathered, allowing the calculation of the cost and responsiveness outcome measures.

The AM service provider’s chief executive officer (CEO) served as the primary source of information concerning the service provider’s processes. Observational data, enterprise resource planning data, and employee interview data were also collected, and video conference interviews were conducted with an engineer at the UMC innovation lab. The transcripts of the taped interviews were then verified by the interviewees, and this information was then used for drafting the current processes (base case; see Appendix A). All the collected data and process maps were validated by the interviewees and used to develop and calculate the future scenarios outlined in the next section.

### B. Scenario Analysis

Scenario analysis [58] can help identify and evaluate realistic future options [59]. In [51], [54], scenario analysis was used to investigate SC optimization by comparing centralized versus localized configurations in an industrial spare parts context. As suggested in [60], we used a three-step approach for strategic SC planning. In the first step, scenarios were built. In the second step, two scenarios were planned (i.e., calculations were made). In the third step, the scenarios were validated by the researcher and by the field representatives before being utilized to generate the results.

Candidate scenarios were identified, and the key influencing factors were determined [60]. We varied the SC configurations between centralized and localized AM production. As the second scenario variable, we utilized the current and future states of technology. In scenarios, the current state of AM technology refers to the use of the EOS P100 SLS machine. The specifications of the Future 1 AM machine are based on the upcoming Formlabs Fuse One AM machine. The specifications of the Future 2 AM machine are also based on the Formlabs Fuse One but with the assumption of dual-chamber automated operation capability to eliminate out-of-machine cooling and the need for cleanroom. It should be noted that the specifications of the Formlabs Fuse One machine are comparable with those of the EOS P100 and that this machine has been verified for medical devices processing and materials. However, it has a smaller production chamber and a less powerful laser [61]. Our six distinct scenarios are presented in Table 1.

**TABLE 1:** Supply Chain Scenarios for the Additive Manufacturing of Patient-Specific Medical Parts

		SC configuration for the Netherlands	
		Centralized	Localized
AM machine state of technology	Current (EOS P100)	Scenario 1 (base case)	Scenario 2
	Future 1 (Formlabs Fuse One)	Scenario 3	Scenario 4
	Future 2 (Formlabs Fuse One with hypothetical capabilities)	Scenario 5	Scenario 6

We also investigated the effects of demand changes, different numbers of service locations, and service distance on the competitiveness of the scenarios using sensitivity analyses. This is detailed in Sections IV.A and IV.B.

In four iteration rounds, the scenarios and calculations were refined and discussed with the AM service provider’s CEO until a final consensus was reached and scenario models were verified. To keep the model simple, possible wastes (e.g., waiting times) were ignored and responsiveness and cost modeling considered delivery to the UMC as the endpoint. Moreover, the inspection and sterilization time (steps 21 and 24; see Fig. 9 in Appendix A) were not included in our model since those are identical in all scenarios. The SC responsiveness or order fulfillment cycle time was calculated using the SC map, and it includes the following steps:

• Digital file preparation: file check, nesting;
• Preproduction: preparation, preheating;
• Production: 3D printing process;
• Cooling: cooling inside and outside the AM machine;
• Postproduction: cleaning, quality check, storage, packaging;
• Transportation (outsourced): shipping of finished goods.

**FIGURE 3.**
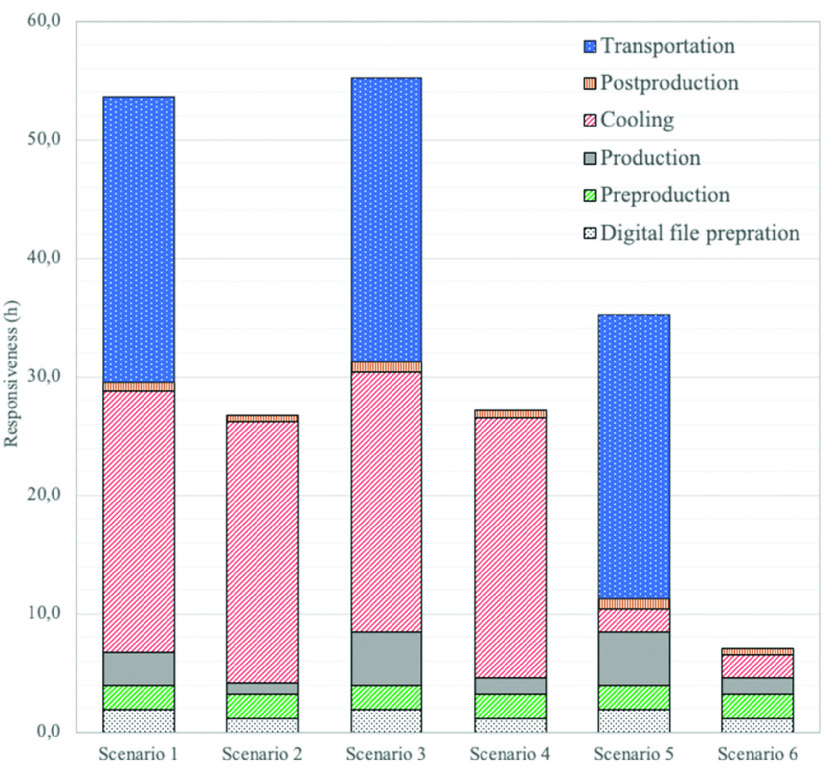
Supply chain responsiveness breakdown.

**FIGURE 4.**
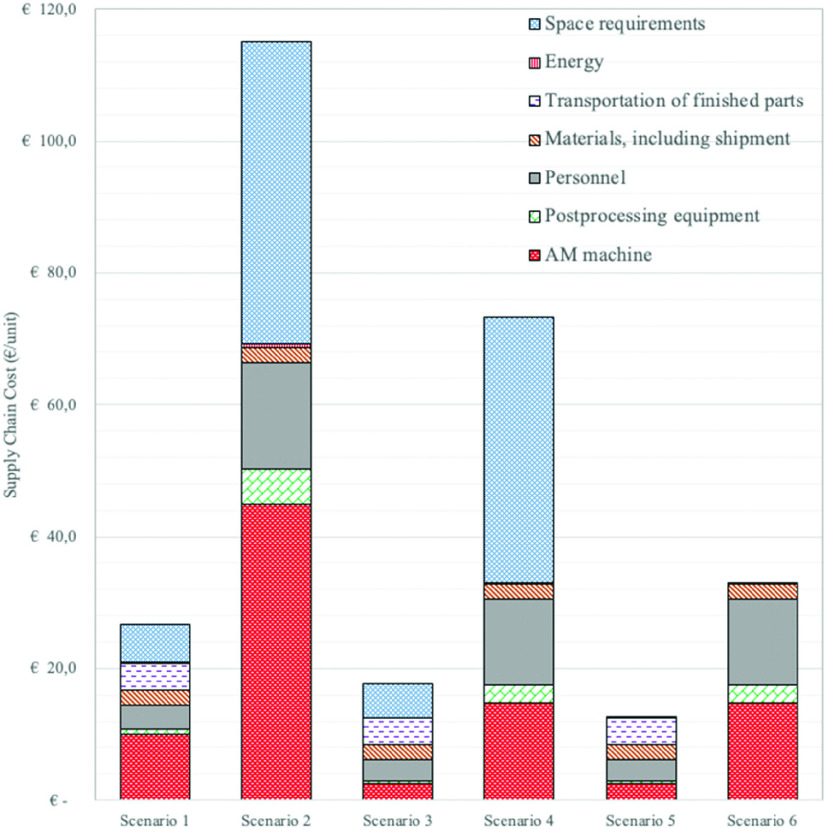
Cost breakdown of the scenarios (€/unit).

**FIGURE 5.**
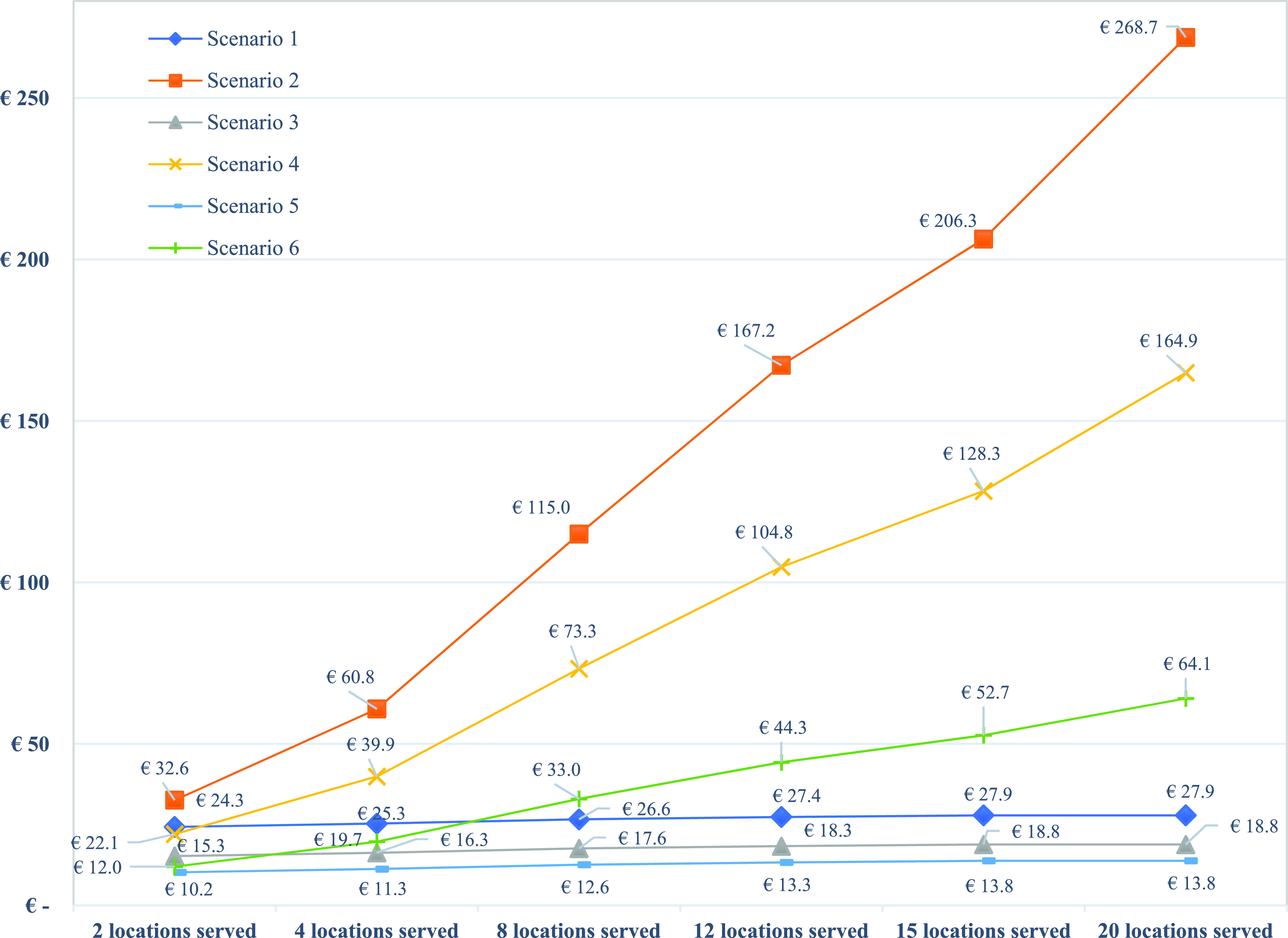
Costs of scenarios for serving different numbers of university hospitals.

**FIGURE 6.**
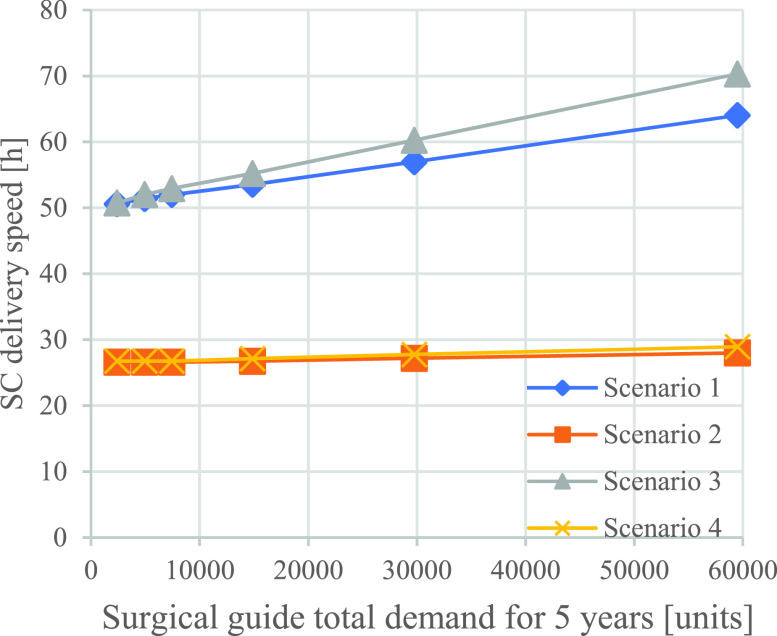
Impact of demand volume on supply chain responsiveness per set (the base case total demand is 14880 units).

**FIGURE 7.**
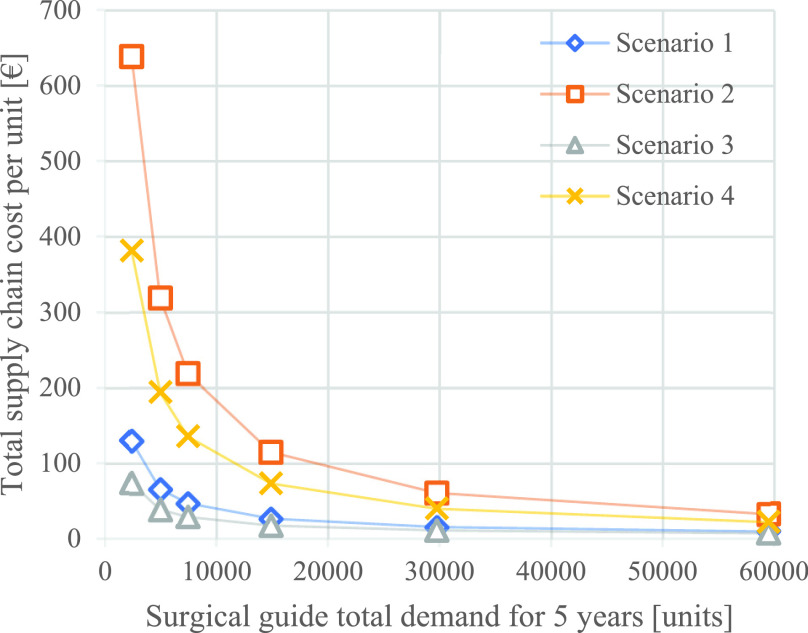
Impact of demand volume on supply chain cost per set (the base case total demand is 14880 units).

**FIGURE 8.**
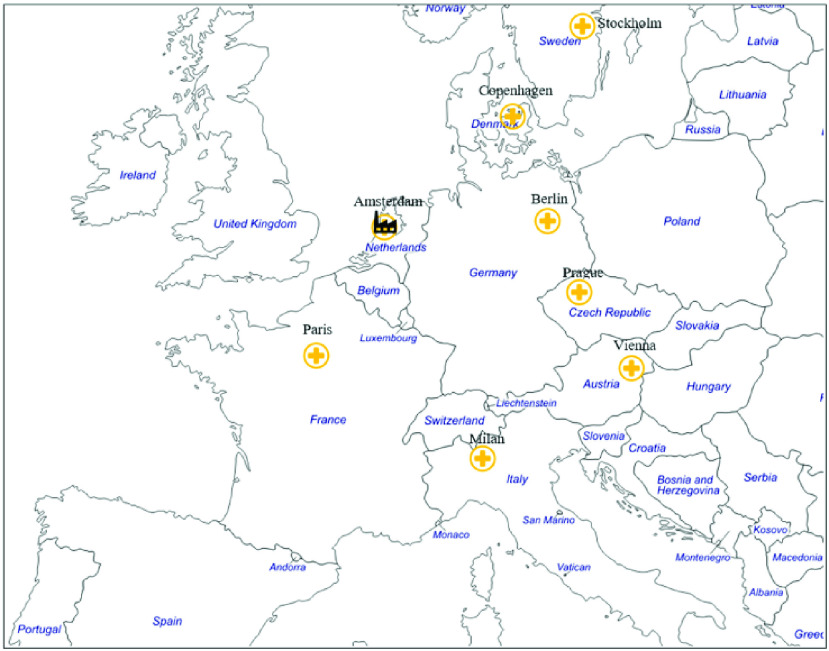
Centralized additive manufacturing of medical parts in Amsterdam to supply other European cities.

**FIGURE 9.**
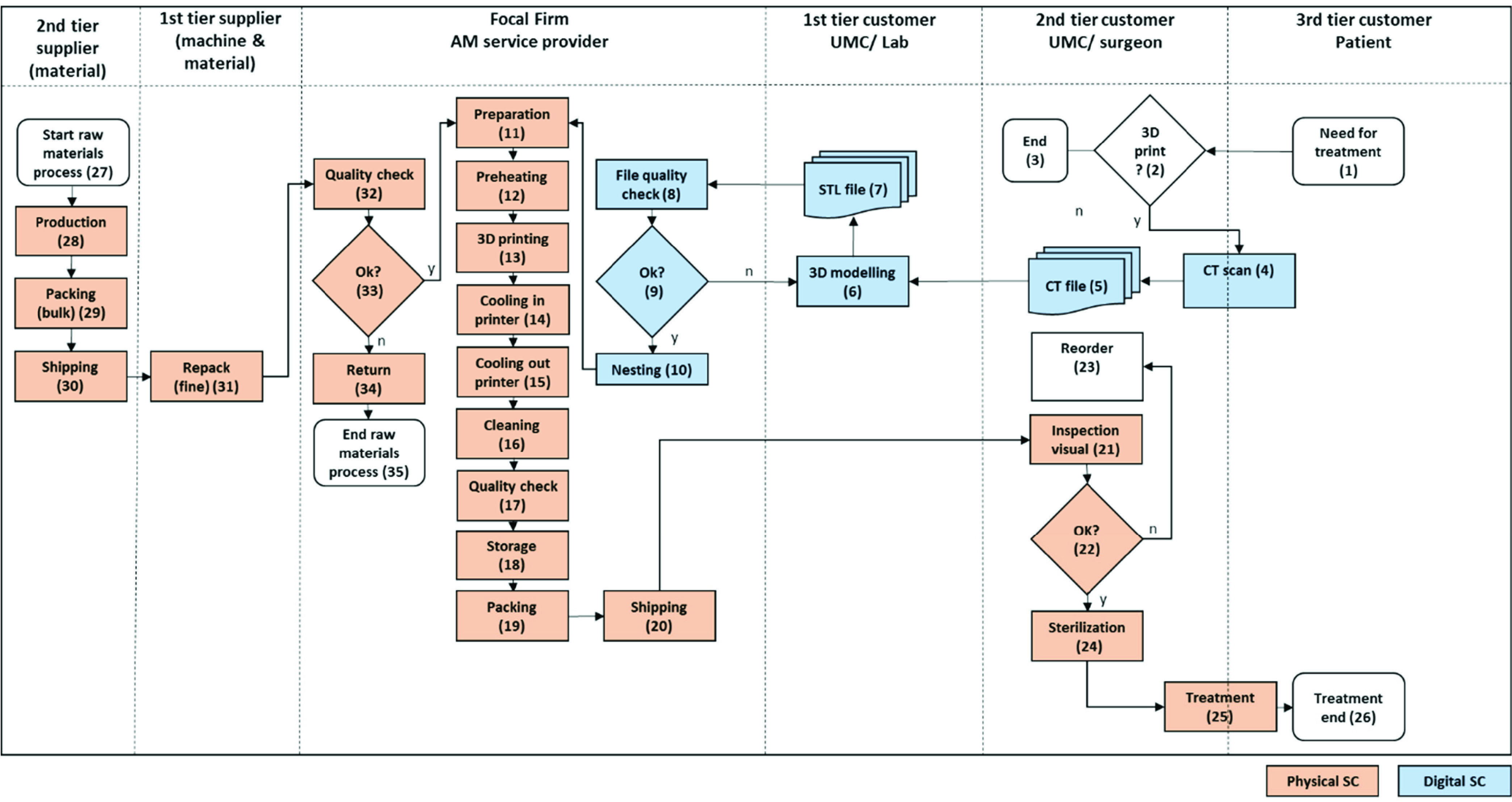
Detailed process chain flowchart of our case study.

The sum of the steps was calculated for the average demand for a period of five years in hours using the formula, as shown at the bottom of the page.

**Figure:** \begin{align*}&\hspace {-1pc}average~order~fulfillment~cycle~of~each~scenario~ \\=&\,\frac {\sum \nolimits _{year 1}^{year 5}time\left ({Digital~file~prepration~,pre\,\,-production~,production~,cooling~, post\,\,-\,\,production~,Transportation~}\right)}{\sum \nolimits _{year 1}^{year 5}number~of~machine~runs}\end{align*}

The following total SC management cost elements were included in our SC cost model:

• Equipment: AM machine investments and maintenance as well as investments in postprocessing equipment (blaster, brush).
• Facilities: facility adaptations (foundation preparation, air conditioning, and air cleaning). Space acquisition or rental costs were excluded.
• Labor: operator training and operational costs, as well as supervisor costs (one supervisor per six operators).
• Material: polyamide raw material.
• Utilities: energy for preheating and manufacturing.
• Transportation (outsourced): finished products from the AM service provider to a UMC.

To allow the comparison of the scenarios, we calculated the cost per part in Euros (€) and used the total SC cost over a five-year period divided by the cumulated five-year volume. The following formula was used to calculate the SC cost, as shown at the bottom of the page.

**Figure:** \begin{equation*} cost~per~part~in~ each~scenario =\,\frac {\sum \nolimits _{year 1}^{year 5}cost\left ({Equipment~,Facilities~,Labor~,Material~,Utilities~,Transportation~ }\right)}{\sum \nolimits _{year 1}^{year 5}Demand~volume}\end{equation*}

Microsoft Excel was used for SC modeling and total cost calculation of the scenarios. For more details regarding the data used for the creation of the scenarios, please refer to Appendix B.

## IV. Results

Section IV.A presents the results of our detailed empirical cost formulation and contributes to a broader agenda related to the development of a decision system. In Section IV.B, we present the findings of the scenario analysis on cost and responsiveness for producing patient-specific medical parts. In Sections IV.C, IV.D, and IV.E, we perform a sensitivity study on important variables, including the number of service locations, demand level, and service distance.

### A. Cost Formulation and Decision Support Parameters

On the basis of our case study, two cost models were formulated to be used by SC practitioners. A centralized AM annual SC cost formulation for the manufacturing of medical parts (EQ1) is presented first:

\begin{align*} {ATC}_{CentAM}=&n_{AM}.\left ({C_{AMM}.{DR}_{AMM}+M_{AMM} }\right) \\&+(C_{PPM}.n_{PPM}.{DR}_{PPM}) \\&+(n_{op}.S_{op}+n_{S}.S_{S}+C_{Tr}) \\&+\left ({C_{M}.{AVGW}_{P}.WR.D }\right) \\&+\left ({C_{E}.E_{AM}.{EXU}_{AMM} }\right) \\&+\left ({{AVGC}_{T}.n_{AT} }\right) \\&+(C_{CR}.A)+C_{i}.n_{AM}\tag{1}\end{align*}

where

${ATC}_{CentAM}$ *is the total annual cost of the centralized AM SC for patient – specific medical parts*;
$n_{AM}$ *is the number of AM machines*;
$C_{AMM}$ *is the purchase price of an AM machine*;
${DR}_{AMM}$ *is the annual depreciation rate of an AM machine, which depends on the utilization rate*;
$M_{AMM}$ *is the annual maintenance rate of an AM machine*;
$C_{PPM}$*is the purchase price of a postprocessing machine (PPM)*;
$n_{PPM}$ *is the required number of PPMs*;
${DR}_{PPM}$ *is the annual depreciation rate of a PPM machine*;
$n_{op}$ *is the number of AM machine operators*;
$S_{op}$ *is the annual salary of an AM machine operator*;
$n_{S}$ *is the number of supervisors*;
$S_{S}$ *is the annual salary of a supervisor*;
$C_{Tr}$ *is the annualized cost of required training*;
$C_{M}$ *is the cost of material per kilogram*;
${AVGW}_{P}$ *is the average weight of the produced parts*;
*WR is the ratio of production waste*;
*D is the total annual demand*;
$C_{E}$ *is the cost of electricity per kilowatt – hour*;
$E_{AM}$ *is the average hourly energy consumption of an AM machine*;
${EXU}_{AMM}$ *is the expected annual utilization hours of an AM machine*;
${AVGC}_{T}$*is the average cost per transportation*;
$n_{AT}$ *is the annual number of shipments*;
$C_{CR}$ *is the cost of constructing 1 m*^2^ *of cleanroom*;
*A is the area of the required cleanroom*;
$C_{i}$ *is the cost of AM machine installation;*

In the cost model for the centralized AM SC, the decision parameters are $\text{n}_{\mathrm {AM}}$, $\text{n}_{\mathrm {PPM}},_{\mathrm {}}~\text{n}_{\mathrm {OP}}$, $\text{n}_{\mathrm {S}}$, $\text{n}_{\mathrm {AT}}$, in addition to the type of AM process that can significantly impact the A, and WR. For instance, a centralized operator can decide to have a larger AM machine so that it can accommodate the growing demand, or perhaps the operator may decide to start with machines with smaller production chambers and increase the number of the machines with the expansion of the business. These decisions impact the short- and long-term competitiveness of a centralized SC against a localized AM SC.

The localized AM annual SC cost formulation for the manufacturing of medical parts (EQ2) is presented as follows:

\begin{align*} {ATC}_{LocalAM}=&n_{L}.{n}_{LAM}.\left ({C_{AMM}.{DR^{\prime }_{AMM}}+M_{AMM} }\right) \\[3pt]&+(C_{PPM}.n_{L}.n_{PPML}.{DR^{\prime }}_{PPM}) \\[3pt]&+n_{L}.(n^{\prime }_{op}.S_{op}+n^{\prime }_{S}.S_{S} +C_{Tr}) \\[3pt]&+\left ({C_{M}.{AVGW}_{P}.WR.D }\right) \\[3pt]&+n_{L}.\left ({C_{E}.E_{AM}.{EXU}_{AMML} }\right) \\[3pt]&+n_{L}.(C_{CR}.A')+{n_{L}.C}_{i}.n_{LAM}\tag{2}\end{align*}

where

${ATC}_{LocalAM}$ *is the total annual cost of the localized AM SC for patient – specific medical parts*;
$n_{L}$ *is the number of locations served with AM*;
$n_{LAM}$ *is the number of AM machines in each local facility*;
$C_{AMM}$ *is the purchase price of an AM machine*;
${DR'}_{AMM}$ *is the annual depreciation rate of an AM machine, considering the average local utilization rate*;
$M_{AMM}$ *is the annual maintenance rate of an AM machine*;
$C_{PPML}$ *is the purchase price of a PPM for each localized facility*;
$n_{PPM}$ *is the required number of PPMs*;
${DR^{\prime }}_{PPM}$ *is the annual depreciation rate of a PPM machine, considering the average local utilization rate*;
$n^{\prime }_{op}$ *is the number of AM machine operators at each localized facility*;
$S_{op}$ *is the annual salary of an AM machine operator*;
${n'}_{S}$ *is the number of supervisors at each localized facility*;
$S_{S}$ *is the annual salary of a supervisor*;
$C_{Tr}$ *is the annualized cost of required training at each facility*;
$C_{M}$ *is the cost of material per kilogram*;
${AVGW}_{P}$ *is the average weight of the produced parts*;
*WR is the ratio of production waste*;
*D is the total annual demand*;
$C_{E}$ *is the cost of electricity per kilowatt – hour*;
$E_{AM}$ *is the average hourly energy consumption of an AM machine*;
${EXU}_{AMML}$ *is the expected average annual utilization hours of AM machines at all locations*;
$C_{CR}$ *is the cost of constructing 1m*^2^ *of cleanroom*;
$A^{\prime }$ *is the average area of the required cleanroom in all local facilities*;
$C_{i}$ *is the average cost of AM machine installation at each local facility*.

In the EQ2 cost model for localized AM SCs for patient-specific medical parts, we can see the main differences with EQ1. The cost of transportation is eliminated because of the local production of demand. Moreover, $\text{n}_{\mathrm {L}}$ is added to the decision parameters of the localized AM SC.

Both EQ1 and EQ2 are among the main empirical contributions of this research, allowing SC practitioners to accurately model the cost of their SC for centralized and localized implementation of AM for patient-specific medical parts. We also determined the parameters that can be tweaked to enhance the decision-making process.

### B. Scenarios

Fig. 3 presents the results of SC responsiveness for the six scenarios. The results show that the localized scenarios (2, 4, and 6) perform better on SC responsiveness, whereas the centralized scenarios (1, 3, and 5) perform better on SC costs (Table 2). It should be noted that such better performance of the localized SC scenarios with regard to responsiveness is mainly due to the elimination of the 24h transportation time between the point of production and demand location. The remaining 27.1h response time of Scenario 4 (localized) is mainly attributed to the 22h cooling time (2h inside plus 20h outside the AM machine) required to ensure product quality and accuracy by reducing warping. It was also observed that the manufacturing time of Scenario 4 is longer than that of Scenario 2 because of the smaller chamber capacity in Formlabs Fuse One and lower laser power. However, the quick responsiveness of Scenario 6 is due to the elimination of the long cooling time outside the machine, enabled by the hypothetical Future 2 AM machine. More information is provided in Appendix C.

**TABLE 2:** Supply Chain Responsiveness and Cost Related to the Different Scenarios

	Scenario					
	1	2	3	4	5	6
Responsiveness (hours)	53.6	26.8	55.3	27.1	35.3	7.1
Cost (€/unit)	26.6	115	17.6	73.3	12.6	33

If a UMC, bearing in mind the technological advances that will be made in the near future using Formlabs Fuse One, has to decide between centralized and localized SC configurations, Scenarios 1 and 4 should be compared. This comparison shows that the unit cost of the localized SC configuration is almost three times higher than that of the centralized SC configuration, with half the SC responsiveness. Moreover, the cost per part in Scenarios 2 and 4 with localized SC configurations is 4.3 and 4.2 times higher than in Scenarios 1 and 3 (centralized AM SC) while using current and future AM machines, respectively. The results show that while the SC responsiveness is slightly reduced with the use of a future AM machine (because of the lower-powered laser in comparison to the current AM machine), the cost competitiveness improves by 57%.

Interestingly, the costs of Scenario 6 are close to those of Scenario 1 (€6.4/unit cost difference). This illustrates the importance of cheaper AM machines with automated internal part cleaning capabilities when considering the cost-effectiveness of localized SCs. Moreover, it was found that Scenario 6 is nearly 46.5h faster than Scenario 1.

Fig. 4 illustrates the major cost components of each scenario and shows that the machine costs are significantly lower for Scenarios 3 and 4 (future AM) than for Scenarios 1 and 2 (current AM). This is due to the high purchase price of the EOS SLS AM machines at €250K.

In general, the costs of the space adjustments are very high in the localized SC scenarios as compared to the centralized SC scenarios. This is mainly due to the high costs required to adjust the eight facilities to prevent the dust dispersion caused by production and postprocessing activities (cleaning and tumbling). The magnitude of this problem becomes more evident when visiting an AM service provider using this SLS/polyamide technology, where the production areas are covered by a thin layer of dust. This is particularly unacceptable in a hospital context, and hence dust-prevention measures are required, such as air-tightening the facilities and installing air-cleaning equipment. In consultation with the AM service provider’s CEO, these facility adaptations are estimated to cost €85K per hospital (including the foundation preparations).

It was also observed that the transportation costs are high in the centralized scenarios, at €3.9/unit, but nonexistent in the localized SC scenarios and that the absolute cost for raw materials (including raw material shipping) is identical, at €2.3/unit, for all six scenarios. Moreover, the personnel costs are significantly lower in the centralized SC scenarios, at €3.7/unit and €3.3/unit, than in the localized scenarios, at €16.2/unit and €12.9/unit, respectively. The reason for such a vast difference is that, in the localized setting, assuming an equal uniform distribution of the demand volume over the locations, the total number of machine runs increases. This causes an increase in the overall number of hours of work. Moreover, having the AM machines in eight different locations means that there is a need for eight times the initial personnel training. The difference in the postprocessing costs between the current and future AM machine scenario results from cutting equipment costs in half, €5K per location, which is then multiplied by the number of locations. In all scenarios, the energy costs have no significant impact on cost performance.

### C. Sensitivity Analysis of the Number of Served Locations

In our case study, there are eight university hospitals that require AM medical parts. However, to understand the impact of larger and smaller numbers of service locations, we performed a sensitivity analysis. In total, two, four, 12, 15, and 20 university hospitals were used for the sensitivity analysis of production cost per part for all six scenarios. As shown in Fig. 5, the three localized scenarios show a larger cost increase than that in the centralized SC scenarios, in which the cost per part increases only slightly. While the cost per part for Scenario 1 increases by only 14.7% when the number of university hospitals increases from two to 20, the cost per part for Scenario 6, which is the most cost-competitive among the localized scenarios, increases by more than 434%. This is mainly due to a large increase in the equipment and facility costs. An interesting finding from this sensitivity analysis is that Scenarios 4 and 6 are cost-competitive with Scenario 1 when two and four university hospitals, respectively, are served (Table 3).

**TABLE 3:** Impact of the Number of Service Locations on the Cost Per Unit of Different Scenarios

		Scenario					
	Cost (€/unit)	1	2	3	4	5	6
No. of service locations	2	24.3	32.6	15.3	22.1	10.2	12
	4	25.3	60.8	16.3	39.9	11.3	19.7
	8	26.6	115	17.6	73.3	12.6	33
	12	27.4	167.2	18.3	104.8	13.3	44.3
	15	27.9	206.3	18.8	128.3	13.8	52.7
	20	27.9	268.7	18.8	164.9	13.8	64.1

### D. Sensitivity Analysis of Demand

To further understand the impact of demand changes on the SC responsiveness and cost, we performed a sensitivity analysis on demand for Scenarios 1 to 4. Figures 6 and 7 illustrate the results of changes in the overall market demand volumes for the demand levels of 2400, 4960, 7440, 14880, 29760, and 59520 units. Increasing the demand does not significantly change the responsiveness of the localized scenarios because of the existence of a significant capacity buffer. However, the responsiveness of the centralized AM SC is reduced by 19.5% and 27.3% for Scenarios 1 and 3, respectively, when the demand quadruples from 14880 to 59520 units. The main source of this reduced responsiveness is the increased production time on the AM machine, since a higher demand requires more parts to be printed during each run, potentially increasing the height of the print and consequently causing longer print times on the available machine.

Fig. 7 shows that the surgical guide set prices drop sharply when the demand increases. When the total demand for a five-year calculation quadruples from 14880 to 59520 units, the unit costs drop by 64% (Scenario 1) and 59% (Scenario 3) in the centralized scenarios. Similarly, the unit costs decrease by 72% (Scenario 2) and 70% (Scenario 4) in the localized scenarios. These high unit costs at low volumes are caused by poor machine and production chamber utilization.

In general, when using the SLS technique, increasing the ratio between the powder and the parts (packing density) is crucial for improving the manufacturing time and cost per part [62]. This results in the need to make as many parts as possible during each production run [63]. In a higher-demand setting, it is possible to take advantage of the empty chamber to produce more parts and, therefore, reduce the overall cost per part. The mechanism underlying this event is that the costs of both pre- and postproduction activities are divided by a larger number in an increased-demand setting. Moreover, in the SLS process, as long as the height of the print job is constant while filling up the chamber, the time required for preheating and recoating each layer is divided by a larger number of parts, which also contributes to a lower cost per part. In summary, higher demand volumes improve the competitiveness of localized AM in comparison to the centralized patient-specific medical parts AM SC configuration in terms of both responsiveness and cost.

### E. Sensitivity Analysis on Distance: Intra-European Service Provision

To improve the generalizability and make our results applicable to large countries, such as the United States, China, Brazil, and Canada, we scaled up the transportation distances to cover some of the main cities of Europe (see Fig. 8). We assume that the manufacturing facility in the Netherlands supplies medical products to UMCs in Amsterdam as well as in Berlin, Paris, Vienna, Copenhagen, Stockholm, Prague, and Milan. Scenarios $1^\prime, 2^\prime, 3^\prime $, and $4^\prime $ were modeled for the intra-European context (Table 4). We extracted the cost and time of intra-European transportation from the webpage of DHL International [64], and we utilized an express delivery service with a delivery time of 24h (delivery at the end of the next day for items posted before 6:00 PM).

**TABLE 4:** Cost Comparison of Centralized and Localized Additive Manufacturing for Medical Parts in the Intra-European Context

	Intra-European			
	Scenario $1^{\prime} $	Scenario $2^{\prime} $	Scenario $3^{\prime} $	Scenario $4^{\prime} $
Cost per part (€)	41.25	115.43	32.23	73.73

Since it was assumed that the demand follows a uniform distribution, the average cost of shipping to the eight destinations has been used. It was found that the average cost is approximately five times higher than the cost of transportation inside the Netherlands (€3.9 versus €18.5). Moreover, as an assumption and to compensate for the longer shipping distances for the localized scenarios, we increased the cost of raw material (which also includes the raw material’s transportation cost) by 20%.

The results showed that, for the intra-European service provision, the responsiveness of the localized scenarios is two times faster than that of the centralized scenarios, which have not changed from the original scenarios (Scenarios 1, 2, 3, and 4). This is because the delivery time between the selected cities is still nearly 24h. However, because of the increased transportation cost, longer distances increase the cost of centralized SCs by 55% and 83% for Scenarios $1^\prime $ and $3^\prime $, respectively. For more details refer to Appendix C.

The findings presented in Table 4 illustrate that the cost competitiveness of the localized AM scenarios significantly increases as the distance between the customer and the point of production increases. It can also be observed that the difference between the total costs of Scenarios 3 and 4 shrinks by 25% when the distance increases. However, Scenarios $2^\prime $ and $4^\prime $ (localized) are still 2.8 and 2.3 times more costly than Scenarios $1^\prime $ and $3^\prime $ (centralized), respectively, for the demand levels used in this research. However, in the case of a pandemic, considering the consequences of lacking medical parts, this might not be a significant cost difference. The major cost components that contribute to the higher total cost of localized AM production are related to the costs of creating a certified space for production, machine cost, and cost of required personnel.

## V. Discussion

In this section, we discuss the implications of the findings, managerial contributions, and generalizability of this study and also point out its limitations.

It has been proven that the SC responsiveness of localized AM is two times higher than that of centralized AM for medical parts while costing 2.3 times more in an intra-European context. Such an extra cost can be justified since the unavailability of time-critical medical or nonmedical products may cause significant losses. In a nonmedical context, this preventable loss is referred to as the equipment downtime cost, and companies generally implement inventory management tools to circumvent this issue, including keeping a safety stock and setting a reordering point. However, when it comes to personalized medical parts, such inventory tools and policies do not apply since there is no stock of patient-specific parts. In this case, the SC responsiveness is related to the process of order placement, production, and transportation.

Interestingly, the results of the sensitivity analysis of the number of service locations (Fig. 5) showed relative cost competitiveness of Scenarios 4 and 6 with Scenario 1 when the number of locations served was only two. When the number of locations increased to 4, Scenario 6 was found to still be cost-competitive with Scenario 1. These findings can be interpreted in three ways. First, using smaller and lower-cost AM machines is preferable for localized AM. Second, the postprocessing and dust-dispersion costs in PBF technologies are a major obstacle for localized SCs and should be addressed to increase the cost competitiveness of localized AM. Third, other technologies, such as fused deposition modeling (FDM), that do not require significant postprocessing may be a potent candidate technology for localized AM of medical parts.

### A. Pandemics and Their Consequences on Centralized Manufacturing

For time-critical medical parts (e.g., parts necessary for a traumatic situation), localized production can reduce the risks of late part delivery by eliminating the shipping time, hence reducing the risk of irreversible damage to people’s lives. This becomes even more pronounced in the case of a pandemic. During a pandemic, similar to the current COVID-19 pandemic, SCs are disrupted because of the difficulties related to production and shipping. In such a setting, a centralized SC is more likely to be disrupted than a localized SC, since it relies on the shipment of items to different cities around a country or to different countries. Therefore, the cost premium paid for establishing a localized SC can be fully justified considering the lower risk of supply interruption and the possibility of increasing the production rate to meet the time-critical local medical demand. In this way, a localized SC may be very suitable for critical medical parts, especially during SC disruption, while also providing a production capacity buffer that can be used in the case of a pandemic [51].

Among the parameters that can improve the cost competitiveness of localized AM are increasing demand and lower AM machine prices coupled with improved pre- and postproduction automation of the AM process. The total cost of a centralized AM SC with a similar affordable and automated AM machine will still be lower than that of a localized SC, but the total cost gap between the two SC configurations decreases.

### B. Generalizability of the Findings and Study Limitations

In general, the findings of this research are transferable to any time-critical AM application under the condition that there is no stock keeping, which is inherent to personalized products. Such a crucial difference between patient-specific surgical guide SCs and industrial spare parts SCs does not allow the findings of this research to be used in applications with stock keeping [56]. However, our findings, modeling, and calculation methods are transferable to contexts without inventory holding, such as the production of other patient-specific medical care products (e.g., molds for implants, splints, and sight models). Moreover, we included two different SC scales in this study: one modeled inside the Netherlands, which is a relatively small country, and the other modeled on a European continent scale, which is comparable to any large country in the world. Therefore, by expanding the analysis to include both large- and small-scale SCs, these research findings can be transferred to large countries, such as the United States, China, Brazil, and Canada.

The fact that this research focuses on a country and a continent that have a well-developed infrastructure and logistics can be considered a limitation of this research. Future research should focus on the comparative competitiveness of AM SC configurations in countries and regions with developing or underdeveloped infrastructures. Moreover, we encourage studies on and comparisons of AM SC configurations for standardized medical components similar to the ones produced during the COVID-19 pandemic, such as nasal swabs, face shield holders, and Venturi valves [2].

Additionally, we recommend that future research address the integral cost savings resulting from reduced OR utilization and hospitalization, as well as employer and societal cost benefits resulting from patient recovery due to localized AM. Here, we investigated the impact of mitigating the challenges rising from the dust dispersion of PBF processes in Scenarios 5 and 6 with promising outcomes. This can be considered an area of open investigation aiming to enhance the (post)production processes of PBF processes and achieve better dust dispersion and faster cooling processes. We also recommend conducting additional research on the use of other AM processes, such as FDM for the localized production of patient-specific medical parts.

### C. Managerial Contributions

Improvements in the cost competitiveness of localized AM SC configurations heavily depend on technological developments and the volume of demand. Therefore, the following are some recommendations for managers and business practitioners:

• To evaluate the cost competitiveness of localized AM SCs with a centralized AM SC for patient-specific medical parts, both EQ1 and EQ2 as well as the decision parameters presented in this research can be utilized.
• When the volume of demand for personalized medical parts increases and more affordable SLS AM machines enter the market, a localized SC can become highly competitive with a centralized configuration, particularly when the shipping methods are unreliable or the distance between the production site and the utilization location is large.
• Developing AM equipment that enables faster production and can offer new solutions for the dust-dispersion issue will allow for the localized use of equipment since it improves the SC responsiveness of localized SCs while also reducing the centralized versus localized SC cost gap. Moreover, AM machines with higher automation in the postprocessing steps can contribute to the competitiveness of localized AM for medical parts because the savings resulting from having fewer AM machine operators will be multiplied by the number of nodes in a localized SC.
• Since high chamber utilization is important to lower the cost of AM, companies can explore various solutions, such as the use of hub configurations (instead of fully self-owned localized AM), to improve the economic feasibility for noncritical medical parts. Moreover, for the production of time-critical medical parts in localized AM, the cost competitiveness can be enhanced by sacrificing an acceptable amount of responsiveness to pack the chamber with other less essential medical parts (e.g., molds for implants, sight models).
• It is worth noting that if a decision to switch from centralized to localized AM is made, centralized AM will continue to exist while localized AM will gradually fade in.

## VI. Conclusion

In general, the results obtained in this study contribute to the limited body of knowledge regarding the decision-making process for SC configurations of additively manufactured medical parts. Such a contribution is achieved by developing two cost formulas based on a real-world case study. Moreover, six scenarios were utilized to shed light on the technological and operational challenges of introducing localized AM to medical centers and hospitals. We investigated the responsiveness and costs of meeting the demand for patient-specific medical parts in centralized and localized AM SC configurations. An empirical cost model was developed for both centralized and localized SCs for producing patient-specific medical parts. The findings showed that, compared to the current centralized SC configuration, the localized scenario with soon-available technology halves the delivery time, but the costs are 4.3 times higher. Accelerated delivery of parts is possible because transportation is no longer necessary in a localized SC configuration. One of the main underlying components contributing to the current AM SC responsiveness is the long cooling-down time needed to prevent product warping. Regarding the major cost components of localized AM, we identified the AM equipment cost and the cost of the measures taken to prevent dust dispersion. Both issues of long cooling time and dust dispersion are due to the SLS AM technology, which can be addressed by the advancement of AM processes and material technologies in the future. Moreover, increasing the demand improves the SC responsiveness and cost competitiveness of localized scenarios compared to centralized ones.

Both the COVID-19 pandemic and the subsequent disruptions in the supply of goods and standard medical equipment illustrated the importance of SC resilience and responsiveness. In particular, when the lack of time-critical patient-specific medical parts causes a stagger in the medical care processes, thus potentially causing irreversible damage to people’s lives, localized AM can be used. Overall, decisions regarding the configuration of AM SCs (centralized versus localized AM SCs) are strongly interrelated with the time criticality of the medical parts, demand volumes, and technological advancements of AM machines. This study shows that although the SC cost of localized AM is higher than that of centralized AM, the lead-time improvement of localized AM is significant. It should be noted, however, that the scope of the present study did not include, for example, insurance- and employer-related issues, such as the potential reduction in sick leaves. Hence, it is safe to say that the potential integral cost savings that can be achieved by accelerating the medical care process may offset any additional unit costs associated with localized AM.

## Appendix

### A. Process Chain Flowchart of Our Case Study

See fig 9.

### B. Scenario Modeling Details

In this appendix, we present the data collected from our real-world case study and the assumptions made for the creation of the scenarios (Tables 5, 6, and 7). We also discuss the calculation method of AM SC responsiveness and the cost for the scenarios. See table 5.

**TABLE 5:** Cost-Related Scenario Data

Category	Description	Unit	Scenario relevance						Remark
			1	2	3	4	5	6	
Equipment	^1^ EOS P100 machine purchase	€250K	y						5 yr service life, €50K resale value
Equipment	EOS P100 machine purchase	€250K		y					10 yr service life (due to much lower utilization rate), €50K resale value
Equipment	EOS P100 maintenance	€5K/yr	y	y				
Equipment	Postprocessing P100 (blaster, brush)	€10K	y	y					5 yr service life, zero resale value
Equipment	^2^ Fuse 1 machine purchase	€20K			y		y		3 yr service life ^3^, zero resale value
Equipment	Fuse 1 machine purchase	€20K				y		y	6 yr service life (due to much lower utilization rate), zero resale value
Equipment	Fuse 1 maintenance	€5K/yr			y	y	y	y	
Equipment	Postprocessing Fuse 1 (blaster, brush)	€5K			y	y	y	y	5 yr service life, zero resale value
Facility	Space preparation (foundation, air conditioner, air cleaner)	€85K	y	y					5 yr service life, zero resale value
Facility	Space preparation (air conditioner, air cleaner)	€75K			y	y			5 yr service life, zero resale value
Labor	Operator cost	€25K/yr	y	y	y	y	y	y	Persons/year, fully loaded
Labor	Supervisor cost	€39K/yr	y		y		y		Persons/year, fully loaded, no supervisor cost for localized scenarios
Labor	Supervisor-to-operator ratio	1:6	y		y		y		Irrelevant for localized scenarios
Labor	Available production hours	1500 h/yr	y	y	y	y	y	y	Includes 85% productivity, sick leaves, and holidays
Labor	Training for EOS P100 FORMIGA operation	3 months	y	y					One-time investment
Labor	Training for Fuse 1 operation	2 weeks			y	y	y	y	One-time investment
Material	PA2200 (PA12, polyamide)	€60/kg	y	y	y	y	y	y	
Utilities	Cost of nonhousehold electricity in the Netherlands	€0.082/kWh	y	y	y	y	y	y	Ref. Eurostat (2018) ^4^
Utilities	Electricity consumption	6 kW/h	y	y					Ref. EOS (2018)
Utilities	Electricity consumption	2 kW/h			y	y	y	y	
Transportation	Forwarder cost per shipping	€10.30	y		y		y		Per daily shipment, constant for all destinations in the Netherlands, maximum runs/year = 260 (equivalent to the number of yearly machine runs)

^1^ EOS P100 FORMIGA SLS AM machine with chamber size 250 $\times$ 200 $\times$ 330 mm

^2^ Fuse 1 SLS AM machine with a chamber size of 165 $\times$ 165 $\times$ 320 mm

^3^ Due to different machine workloads in different configurations.

^4^ The value € 0.082/kW/h used in the calculation deviates from that in [65], with an insignificant influence on the results.

**TABLE 6:** Supply Chain Responsiveness-Related Scenario Data

Step no.	Process step description	Unit	Scenario relevance						Remark
			1	2	3	4	5	6	
8	File check	5 min	y	y	y	y	y	y	Per part
9	File check	0	y	y	y	y	y	y	Decision
10	Nesting	1 h	y	y	y	y	y	y	1–50 units, 1 h; +50 units, 1.5 h
11	Preparation	0	y	y	y	y	y	y	
12	Preheating	2 h	y	y	y	y	y	y	Per batch
13	3D printing (only the manufacturing step)	2.8 h	y						^1^ Per batch, note: build height and surface fullness dependent
13	3D printing (only the manufacturing step)		0.97 h		y				^2^ Per batch, note: build height and surface fullness dependent
13	3D printing (only the manufacturing step)	4.47 h			y				Per batch, note: build height and surface fullness dependent
13	3D printing (only the manufacturing step)	1.35 h				y			Per batch, note: build height and surface fullness dependent
13	3D printing (only the manufacturing step)	4.47 h					y		
13	3D printing (only the manufacturing step)	1.35 h						y	
14	Cooling inside the machine	2 h	y	y	y	y	y	y	Per batch
15	Cooling outside the machine	20 h	y	y	y	y			Per batch
16	Cleaning	0.6 h	y		y		y		30 min/batch +30 s/piece
16	Cleaning	0.52 h		y		y		y	30 min/batch +30 s/piece
17	Quality check	0	y	y	y	y	y	y	Integrated into the cleaning process
18	Storage	0	y	y	y	y	y	y	Neglected waste
19	Packing	0.19 h	y		y		y		1 min per piece
19	Packing	0.04 h		y		y		y
20	Shipping	24 h		y		y		y	Contracted out

^1^ The formula takes the production layer thickness and layer fullness into account. The production layer thickness is assumed to be 0.1 mm for all 3D printers. The formula also calculates the initial empty layers (20 layers in this study) below the parts and empty layers above the parts (20 layers in this study). Moreover, the heating, scanning, and recoating of each layer are the three main elements of time calculation in the formula. The assumption for a full layer on EOS P100 is that preheating (during the production), laser scanning, and powder recoating take 16 s for recoating and layer heating and 80 s for laser scanning, depending on the fullness of each layer. All the 3D printing manufacturing step calculations are performed for the average demand level over a five-year production period.

^2^ The formula takes the production layer thickness and layer fullness into account. The production layer thickness is assumed to be 0.1 mm for all 3D printers. The formula also calculates the initial empty layers (20 layers in this study) below the parts and empty layers above the parts (20 layers in this study). Moreover, the heating, scanning, and recoating of each layer are the three main elements of time calculation in the formula. The assumption of a full layer on Future 1 (Fuse 1) and Future 2 is that preheating (during the production), laser scanning, and powder recoating take 16 s for recoating and layer heating and 70 s for laser scanning, depending on the fullness of each layer. All the 3D printing manufacturing step calculations are performed for the average demand level over a five-year production period.

**TABLE 7:** Volume-Related Scenario Data

Description	Unit	Scenario relevance						Remark
		1	2	3	4	5	6	
Number of surgical guide sets (start volume)	240/yr	y	y	y	y	y	y	^1^ A set consists of two individual surgical guides
^2^ Number of UMCs	8	y	y	y	y	y	y	Uniform distribution (in time and volume)
Maximum number of production runs per AM machine	260/yr	y	y	y	y	y	y	Five working days $\times52$ weeks
^3^ Surgical guide weight (average)	35g/unit	y	y	y	y	y	y	Excluding 10% waste
Surgical guide dimensions	$150\times 50\times10$ mm	y	y	y	y	y	y	—

^1^ Each set of surgical guides consists of several parts. In the calculations, we assumed that each set, on average, consists of two parts. The base case scenario starts with a volume of 240 sets, equal to 480 units/year. Assuming that this doubles yearly, this leads to a cumulative five-year demand of 14880 units.

^2^ There are eight UMCs in the Netherlands. A UMC is a hospital affiliated to a medical faculty of a university and combines regular patient care tasks with scientific research and education of medical professionals.

^3^ The average weight of the surgical guides is set at 35 g/unit resulting in a raw material cost of €2.3/unit.

See table 6.

See table 7.

### C. Supply Chain Responsiveness and Cost Component Calculation of the Scenarios

In this appendix, we present a breakdown of our responsiveness (Table 8) and cost (Table 9, 10) calculation results for our centralized and localized AM SC scenarios.

**TABLE 8:** Supply Chain Responsiveness Breakdown Related to the Different Scenarios

Dutch context						
Scenario						
^1^ Responsiveness components	1	2	3	4	5	6
Digital file preparation $(h)$	2	1.2	2	1.2	2	1.2
Preproduction $(h)$	2	2	2	2	2	2
Production $(h)$	2.8	1	4.5	1.3	4.5	1.3
Cooling $(h)$	22	22	22	22	2	2
Postproduction $(h)$	0.8	0.6	0.8	0.6	0.8	0.6
^2^ Transportation $(h)$	24	0.0	24	0.0	24	0.0
Total time $(h)$	53.6	26.8	55.3	27.1	35.3	7.1

^1^ In the responsiveness calculations, we excluded the process wastes (e.g., waiting times) and only used the net time. We also excluded from the calculations the waiting time for batch completion before nesting and production. Sterilization of the products is required before they can be used in the operating theater. This is performed internally at the UMCs (in batches) and requires approximately 2 h. We did not include this step in our cost and responsiveness calculations.

^2^ According to the contract, the delivery of the products should take place within 24 h, although in reality the delivery time is often shorter. In our research, however, we specified 24 h delivery as the only type of delivery in the centralized scenarios. Generally, this has a negative impact on the feasibility of a localized SC configuration as it eliminates the existence of costly but necessary emergency delivery.

**TABLE 9:** Cost Per Part Related to the Different Scenarios

Dutch context						
Scenario						
^1^ Cost components	1	2	3	4	5	6
AM machine (€)	10.1	44.8	2.6	14.9	2.6	14.9
Postprocessing equipment (€)	0.7	5.4	0.3	2.7	0.3	2.7
Personnel (€)	3.7	16.2	3.3	12.9	3.3	12.9
Materials, including shipment (€)	2.3	2.3	2.3	2.3	2.3	2.3
^2^ Transportation of finished parts (€)	3.9	—	3.9	—	3.9	—
Energy (€)	0.2	0.6	0.1	0.2	0.1	0.2
Space requirements (€)	5.7	45.7	5.0	40.3	0.0	0.0
Cost (€/unit)	26.6	115.0	17.6	73.3	12.6	33.0

^1^ Systems enabling safe and efficient sharing of data files are required. However, it was not possible to include these elements in our modeling and calculations.

^2^ The centralized scenarios employ subcontracted transportation with daily pick-up and a 24 h delivery service, according to the shipping agreement. For emergency surgeries, which comprise 20% of the orders, this is unlikely to be optimal, and immediate pick-up (taxi service straight to the lab, bypassing the front desk) may reduce the transportation time to approximately 2 h. This may, however, come at a high cost, approximately €200 per order, and was therefore excluded from the calculations.

**TABLE 10:** Cost Comparison of Centralized and Localized Medical Parts Additive Manufacturing for Four Main Scenarios in the Context of Intra-European Operations

	Intra-European context			
Cost components	Scenario $1^{\prime} $	Scenario $2^{\prime} $	Scenario $3^{\prime} $	Scenario $4^{\prime} $
AM machine (€)	10.1	44.8	2.6	14.9
Postprocessing equipment (€)	0.7	5.4	0.3	2.7
Personnel (€)	3.7	16.2	3.3	12.9
Materials, including transportation (€)	2.3	2.8	2.3	2.8
Transportation of finished parts (€)	18.5	—	18.5	—
Energy (€)	0.2	0.6	0.1	0.2
Space requirements (€)	5.7	45.7	5	40.3
Cost (€/unit)	41.2	115.5	32.1	73.8
